# Shape-Sensing Robotic-Assisted Bronchoscopy vs. Electromagnetic Robotic-Assisted Bronchoscopy—A Comparative Cohort Study

**DOI:** 10.3390/jcm15031284

**Published:** 2026-02-05

**Authors:** See-Wei Low, Fatima Abdeljaleel, Brett Kemper, Yifan Wang, Xiaofeng Wang, Christopher Yurosko, Graham Stockdale, Colin Gillespie, Thomas Gildea, Sonali Sethi, Joseph Cicenia, Michael Machuzak, Francisco Almeida, Bryan S. Benn

**Affiliations:** 1 Department of Pulmonary Medicine, Cleveland Clinic, Cleveland, OH 44195, USAsethis@ccf.org (S.S.);; 2 Department of Internal Medicine, Cleveland Clinic, Fairview Hospital, Cleveland, OH 44111, USA; 3 Department of Internal Medicine, Cleveland Clinic Main Campus, Cleveland, OH 44195, USA; 4 Department of Quantitative Health Sciences, Cleveland Clinic, Cleveland, OH 44195, USA; 5 Departments of Pulmonary Medicine and Critical Care Medicine, Cleveland Clinic, Cleveland, OH 44195, USA

**Keywords:** lung nodule, peripheral pulmonary lesion, navigation bronchoscopy, robotic bronchoscopy, diagnostic yield

## Abstract

**Introduction**: Lung cancer is a leading cause of cancer-related deaths globally, with approximately 1.5 million new peripheral pulmonary lesions (PPLs) detected annually in the United States. Robotic-assisted bronchoscopy (RAB) has emerged as a promising technology, with two platforms initially approved, the Monarch platform (Auris Health Inc, Redwood City, CA, USA) and the Ion Endoluminal System (Intuitive Surgical, Sunnyvale, CA, USA), offering improved stability and distal airway visualization. As RAB adoption increases, there is a critical need for comparative effectiveness data of these systems to guide clinical decision-making and institutional investments. This study aims to compare the diagnostic yield and safety profiles of the Ion and Monarch RAB platforms after introduction at a single institution. **Methods**: We conducted a retrospective chart review of patients undergoing RAB in the first six months following the introduction of each platform. Demographic and radiographic data were collected. Diagnostic yield was defined as obtaining a malignant or specific benign diagnosis from bronchoscopy. **Results**: The study included 56 Ion and 36 Monarch procedures. Diagnostic yield was similar between Ion (75%) and Monarch (72%) groups (*p* = 0.8), with an adjusted odds ratio 0.89 (95% CI 0.30–2.72). Complications were low, with one pneumothorax occurring in each group. **Conclusions**: Early adoption and use of both RAB platforms suggests comparable diagnostic yields and safety profiles in our limited sample size. Larger studies including standardized anesthesia protocol and systematic use of real-time imaging are needed for further evaluation and comparative analysis.

## 1. Introduction

Lung cancer remains the leading cause of cancer death in the United States [1]. Early detection improves outcomes, and widespread imaging now identifies approximately 1.5 million new pulmonary nodules annually [2]. Unfortunately, only a minority of these nodules are early, potentially curable cancers, hence creating a pressing need for accurate and safe tissue diagnosis of peripheral pulmonary lesions (PPLs), particularly for patients with risk factors or in regions with a higher prevalence of malignancy [3]. While bronchoscopic approaches are guideline-supported for patients to obtain a diagnosis [4], they have historically shown variable yield, hence the evaluation of newer navigation technologies using robotic assisted bronchoscopy (RAB) with potential advantages over prior technologies [5,6,7,8,9,10,11].

The first RAB system, the Monarch platform by Auris Health (Johnson & Johnson MedTech, Redwood City, CA, USA), utilizes ENB-based technology and was approved in 2018. Subsequently, shape-sensing RAB with the Ion Endoluminal System by Intuitive Surgical (Intuitive Surgical, Sunnyvale, CA, USA) was approved in 2019. A third system, Galaxy by Noah Medical (Noah Medical, San Carlos, CA, USA), was also recently approved. These platforms, in which the bronchoscopist controls the insertion and articulation of the robotic bronchoscope or catheter with a controller near the patient, offer improved stability of the catheter and visualization of distal airways compared to a standard bronchoscope or older generations of ENB. Initial studies using RAB with ENB [12,13] or with Ion [14,15,16] are promising, and suggest comparable outcomes between the platforms, but comparison across published data is challenging as prior studies used different definitions of diagnostic yield, included different patient populations and levels of operator experiences, and utilized various secondary confirmation tools. However, a recent single-center retrospective study demonstrated a higher diagnostic yield and shorter procedure time for Ion compared to Monarch with the use of real-time image updates using mobile cone-bean CT [17].

As RAB technologies quickly gain popularity in diagnostic bronchoscopy, there is a risk that they are being implemented without adequate supporting evidence. It is essential to have comparative data to guide patient care and inform health-system capital investments. Therefore, we sought to compare the diagnostic yield and safety profile of the Ion and Monarch platforms after their introduction at our institution, adding comparative data to this rapidly evolving field.

## 2. Methods

### 2.1. Data Collection

Study participant demographic information including sex, age, and smoking history was collected by chart review. Radiographic information included lesion location, size, and presence or absence of a radiographic bronchus sign, based on review of the electronic medical record (including bronchoscopy procedure note, radiology report, and available imaging). When needed, discrepancies were adjudicated by two physicians (SWL and BSB). We applied a strict diagnostic yield definition that excluded non-specific benign diagnoses (e.g., normal lung or airway, non-specific inflammatory changes, and atypia not definitely diagnostic of malignancy) determined from the finalized pathology results of the bronchoscopy [18,19]. A procedure was considered diagnostic when a malignant or specific benign diagnosis (e.g., granulomatous inflammation, inflammation confirmed with microbiology data) was made at the index bronchoscopy procedure and readily explained the presence of the PPL to guide further management in the patient’s care. This study received institutional review board approval from the Cleveland Clinic Foundation (#IRB 23-195).

### 2.2. Procedures

This is a single-center, retrospective study reviewing consecutive adult patients who underwent RAB biopsy of a single-target PPL using either RAB platform during the first six months after the introduction of Monarch in August 2021 and Ion platform in March 2022. Only one PPL per patient was biopsied during the procedure and no patients in this cohort were a repeat biopsy. Data from the first six months after the introduction of each platform were obtained to minimize bias from operator experience.

All procedures were performed by board-certified interventional pulmonologists or an interventional pulmonology fellow under the direct supervision of a board-certified interventional pulmonologist. All procedures were conducted in a dedicated bronchoscopy suite under general anesthesia provided by a certified registered nurse anesthetist supervised by an anesthesiologist. Anesthetic care in all procedures consisted of total intravenous anesthesia with neuromuscular blockade and endotracheal intubation with an 8.5 mm endotracheal tube. At our institution, the guidelines set for peripheral navigation cases include the use of positive end expiratory pressure (PEEP) at 8–12 cm of H2 per bronchoscopist’s discretion, tidal volume of 10–12 mL/kg, and variable fraction of inspire oxygen (FiO_2_) titrated to maintain an oxygen saturation of >92%.

Radial endobronchial ultrasonography (rEBUS) was available for use in all patients for both platforms, and all biopsies were performed under fluoroscopic guidance with three-dimensional (3D) imaging capability (Cios Spin by Siemens Healthineers, Malvern, PA, USA). In the Ion group, the cases were performed without integration of the 3D imaging into the Ion system. However, 3D spin with reconstruction could be performed and marked on the fluoroscopy unit for visualization during the procedure at the discretion of the bronchoscopist. Similarly, the LungVision System (Body Vision Medical Inc., Waltham, MA, USA) was available for use at the discretion of the bronchoscopist for Monarch procedures. Transbronchial needle aspiration (TBNA) was the initial biopsy method in all patients. Initial passes were submitted for rapid on-site cytologic examination (ROSE) in all cases. Additional sampling methods (forceps, cytology brush, focused peripheral wash) were performed after TBNA at the operator’s discretion. Procedural data and complications were recorded retrospectively.

### 2.3. Outcomes

The primary outcome was the diagnostic yield between the two robotic navigation platforms. Secondary outcomes included factors associated with diagnostic yield and the rate of complications. The secondary outcomes included radial ultrasound image acquisition (concentric vs. eccentric), nodule size, nodule location, and nodule density.

### 2.4. Statistical Analysis

The demographic and procedure characteristics of patients were compared between the two robotic bronchoscopy platforms, Ion and Monarch. Univariable and multivariable logistic regression analyses were conducted to evaluate the associations between clinical characteristics and diagnostic yield. The clinical characteristics included robotic platforms (Ion vs. Monarch), radial ultrasound image acquisition (concentric vs. eccentric), nodule size, nodule location, and nodule density. A *p*-value < 0.05 was considered statistically significant. All analyses were performed using R statistical software (version 4.3.1).

## 3. Results

In the first six months after each platform’s introduction, Ion was utilized for 56 PPL biopsies, while Monarch was used for 36 PPL biopsies. The groups were well-balanced for most demographic and radiographic features (Table 1 and Table 2). However, the median lesion size significantly differed, with a median of 24 mm (interquartile range, 16–30 mm) for the Ion group and 16 mm (interquartile range, 11–26 mm) for the Monarch group (*p* = 0.029). Sixty-three percent of PPLs were located in the peripheral lung third in the Ion group (35/56) compared to 36% in the Monarch group (13/36, *p* < 0.03), while more nodules were in the middle one-third of the lung in the Monarch group (47%, 17/36) compared to the Ion group (23%, 13/56). A 3D spin was performed in 16% (9/56) of Ion procedures, while the LungVision navigational system was used in 5.6% (2/36) of Monarch procedures, with no 3D spins being performed during Monarch cases.

The primary outcome of diagnostic yield was similar between the Ion (75%, 42/56) and Monarch (72%, 26/36) groups (*p* = 0.8) (Table 3). Among patients undergoing biopsy with Ion, 68% of PPLs (38/56) were malignant, and 7% (4/56) showed specific benign histopathologic features. Among patients undergoing biopsy with Monarch, 69% of PPLs (25/36) were malignant, and 3% (1/36) showed specific benign histopathologic features. In both univariable and multivariable analyses, none of the clinical characteristics were significantly associated with diagnostic yield (all *p* > 0.05). Specifically, the crude odds ratio (OR) for Monarch compared to Ion was 0.87 (95% CI: 0.34–2.28; *p* = 0.77). After adjusting for other characteristics, the adjusted odds ratio (aOR) remained similar at 0.89 (95% CI: 0.30–2.72; *p* = 0.83), indicating no meaningful difference in diagnostic yield between the two robotic platforms (Table 4).

The incidence of complications was rare, with pneumothorax occurring in one patient in each of the Ion and Monarch groups, with both patients requiring tube thoracostomy. Additionally, one patient required advanced intervention (instillation of cold saline) for bleeding in the Ion group. There were no reported complications secondary to bleeding in the Monarch group.

## 4. Discussion

In this single-center retrospective comparative cohort study we found no meaningful difference in diagnostic yield for PPL biopsy between Ion and Monarch platforms during the first six months after the implementation of each system. Using a strict diagnostic yield definition aligned with contemporary consensus recommendations, both platforms achieved yields in the low-to-mid 70% range, with rare complications. These findings add pragmatic comparative data to a rapidly expanding area where technology adoption has outpaced the availability of standardized outcomes and head-to-head evidence. The historically limited diagnostic yield from bronchoscopic biopsies of PPLs is due to multiple factors. CT to body divergence, or the difference between the virtual bronchoscopic target based on CT chest imaging obtained prior to the procedure and the real-time location of the PPL in the patient, have been shown to significantly impact procedural results [20,21]. Additionally, challenges exist in advancing the bronchoscope into smaller peripheral airways due to size limitations as subsegmental bronchi become progressively smaller. Subsegmental bronchi often branch at varying angles, which may be difficult to negotiate with the use of conventional bronchoscopes or legacy navigation platforms. These difficulties in reliably establishing a definitive diagnosis present a significant obstacle in the clinical management of patients with suspected lung cancer [11,22]. Recent advances in anesthesia ventilatory strategy may also positively impact bronchoscopic biopsies and the yield. Efforts to prevent atelectasis include a lower fraction of inspired oxygen (FiO_2_) of 0.6 to 0.8 for pre-oxygenation and maintained at the lowest tolerable level for the entire procedure in addition to the use of positive end expiratory pressure (PEEP) of up to 10–12 cm H_2_O and increased tidal volumes may help to maintain optimal lung inflation, if these settings are tolerated by the patient as determined during lung recruitment [23,24].

RAB systems seek to overcome many of the identified challenges in bronchoscopic biopsies of PPLs. By incorporating shape sensing (Ion) and ENB (Monarch) technologies, the small diameter of catheters in RAB systems aim to improve diagnostic accuracy through precise navigation into small, distal airways with stable catheter positioning and the ability to angulate the catheter in a precise orientation for biopsy tool deployment while maintaining a comparable safety profile. Differences in the virtual bronchoscopy navigation planning software may also play a role. An initial study comparing PPLs on 25 chest CTs showed a statistically significant difference in the distance from the terminal end of the virtual navigation pathway to the target PPL and differences in the generation of complete distal airway maps based on the platform utilized [25].

Recent meta-analyses support an improved diagnostic yield for RAB biopsies of PPLs. In comparison to historical results for the diagnostic accuracy of ENB biopsies of PPLs ranging from 65 to 74% [8,26], recent meta-analyses report the diagnostic yield of RAB ranges from 80 to 84% [27,28]. A recent comparative study of DT-ENB and Ion showed similar diagnostic yields of 80% and 77%, respectively, (*p* = 0.04) [29]. Similarly, a recent meta-analysis supports an increased diagnostic yield for PPLs biopsied by RANB or DT-ENB (77.5% (95–CI 74.7–80.1%)) compared to ENB or virtual bronchoscopy (68.8% (95–CI 65.9–71.6%), *p* < 0.001) [30]. A comparative retrospective study of the two RAB platforms showed higher diagnostic yield and shorter procedural time with Ion compared to Monarch with high utilization of 3D real-time imaging [17]. In our cohort, neither platform used integrated real-time three-dimensional imaging correction within the robotic system (and advanced confirmation tools were used infrequently), which may have limited the ceiling yield for both platforms and reduced the opportunity for platform-specific differences to emerge. Our findings do not contradict those data; rather, they suggest that when both platforms are implemented early and used without routine integrated intraprocedural correction, yields may converge. While these studies are informative, a lack of a unifying definition for “diagnostic yield” across them hinders the comparison of results. A recent Delphi consensus statement proposed using a strict diagnostic yield definition to standardize our current and future research framework [19].

In our study, we compared diagnostic yield from all procedures performed within the first six months using the Monarch platform and the first-generation Ion platform without integrated or intra-procedural real-time imaging correction for CT to body divergence. We utilized the recommended strict diagnostic yield definition in our study, where the numerator included all patients with PPLs in whom the result of a diagnostic procedure establishes a specific benign or malignant diagnosis that is sufficient to inform patient care, and the denominator included all patients in whom the procedure was attempted or performed [19,29]. Diagnostic yield of both platforms was comparable (Ion = 75%, 42/56 vs. Monarch 72%, 26/36, *p* = 0.8) (Table 3), with similar results compared to prior meta-analyses. We believe that our findings likely reflect the improved catheter stability, visual-based navigation through the peripheral airways guided by accurate robotic technology, and the ability to adjust catheter articulation more accurately during target lesion biopsy facilitated by RAB-based systems. Our yield was lower than some single-center RAB reports [14,31], and this may reflect the absence in our procedures of additional real-time 3D intraprocedural imaging for secondary confirmation and potentially the lack of adoption to the more recent anesthesia protocol for peripheral bronchoscopy. Additional studies comparing the diagnostic yield of procedures with and without 3D intraprocedural imaging may be informative to further clarify the impact of this modality. Lesion features differed between groups, including smaller median lesion size in the Monarch cohort and a higher proportion of outer-third lesions in the Ion cohort. Both smaller size and more peripheral location are recognized drivers of lower bronchoscopic yield; thus, these imbalances could bias comparisons in either direction depending on which characteristic predominates clinically. While multivariable modeling did not identify significant associations with yield, the confidence intervals were wide, and the study may be underpowered to detect modest but clinically relevant differences, especially if platform effects interact with lesion size, density, or rEBUS view.

In our early experience, complications were uncommon with either RAB platform. Pneumothorax requiring tube thoracostomy occurred in one patient in both groups (Table 3). Additionally, one patient in the Ion group required intervention for bleeding. These complication rates are comparable to other guided bronchoscopy studies [16,29,32] and improved compared to TTNA [32,33]. The favorable safety profile of RAB may be attributable to catheter stability and preprocedural planning that helps demarcate pleural boundaries and plan trajectories that avoid pleural boundaries. Our study has several limitations. As a non-randomized study with a limited sample size, unmeasured confounding factors may have influenced the results, and statistical power may be insufficient to detect small differences in diagnostic yield, despite multivariable adjustment. As a single-center study, results may not be fully generalizable, particularly because the RAB rollout at our institution predated implementation of a standardized ventilatory strategy. In addition, platform scheduling depended on bronchoscopist and patient availability, resulting in unequal sample sizes and potential differences in case selection. Despite these limitations, strengths of this retrospective study include well-defined criteria for a diagnostic procedure and the lack of exclusion of any case from the analysis owing to run-in time, making the results more generalizable. While the sample size is relatively small, this comparative study between the two initial RAB platforms adds more data to the evolving field of bronchoscopic PPL biopsy. Given the wide confidence intervals and the absence of significant associations, future studies with larger sample sizes may be necessary to achieve sufficient statistical power to detect potential meaningful differences in diagnostic yield among these clinical characteristics.

In conclusion, with the use of strict definitions of diagnostic yield, our study suggests a comparable diagnostic yield and safety profile for RAB with Ion or Monarch. Future randomized trials of RAB platforms will be needed to evaluate these findings and assist in identifying a specific patient population or PPL feature that may predict a higher likelihood of obtaining a confident diagnosis with these platforms.

## Figures and Tables

**Table 1:** Demographic data.

Variables	Ion Shape-Sensing RAB, N = 56	Monarch RAB, N = 36	*p* Value
Age, years	70 ± 11.2	66 ± 12.2	0.10
Gender			>0.9
Male	24 (43)	15 (42)	
Female	32 (57)	21(58)	
Smoking status			0.2
Current	18 (32)	6 (17)	
<30 Packyears	1 (6)	0	
>30 Packyears	17 (94)	6 (100)	
Former	29 (52)	25 (69)	
<30 Packyears	21 (72)	9 (25)	
>30 Packyears	8 (27)	16 (64)	
Never	9 (16)	5 (14)	

Data are presented as No. (%) or mean ± standard deviation, unless otherwise indicated. RAB = robotic-assisted bronchoscopy.

**Table 2:** Comparison between Ion and Monarch lesion and procedure characteristics.

Nodule Characteristics	Ion Shape-Sensing RAB, N = 56	Monarch RAB, N = 36	*p* Value
Lesion size, mm	24 (16–30)	16 (11–26)	0.029
Lesion location			0.032
Peripheral third	35 (63)	13 (36)	
Middle third	13 (23)	17 (47)	
Central	8 (14)	6 (17)	
Specific lobes			0.5
Right upper lobe	24 (43)	12 (33)	
Right middle lobe	6 (11)	3 (8)	
Right lower lobe	9 (16)	3 (8)	
Left upper lobe	11 (20)	12 (33)	
Left lower lobe	4 (7)	5 (14)	
Lingula	2 (3)	1 (2)	
Nodule density			0.3
Solid	52 (93)	31 (86)	
Part solid	4 (7)	5 (14)	
Ground glass opacity	0	0	
Radial ultrasound image			0.2
Concentric	31 (55)	15 (43)	
Eccentric	24 (43)	17 (49)	
No view	1 (1)	3 (8)	
Missing data	0	1	
Presence of bronchus sign	38 (68)	29 (81)	0.2
Presence of emphysema radiologically	20 (36)	17 (47)	0.3

Data are presented as No. (%) or median (interquartile range), unless otherwise indicated. RAB = robotic-assisted bronchoscopy.

**Table 3:** Diagnostic findings for Ion and Monarch cohorts.

	Ion Shape-Sensing RAB, N = 56 (%)	Monarch RAB, N = 36 (%)
Diagnosis yield		
Diagnostic	42 (75)	26 (72)
**Malignant**	38 (67.9)	25 (69.4)
NSCLC adenocarcinoma	11 (28.9)	12 (48)
NSCLC squamous cell carcinoma	15 (39.5)	3 (12)
NSCLC, NOS	6 (15.8)	5 (20)
Small cell carcinoma	1 (2.6)	1 (4)
Carcinoid tumor	3 (8.0)	1 (4)
Metastatic disease from other solid tumors	1 (2.6)	2 (8)
Lymphoma	0	1 (4)
Poorly differentiated carcinoma	1 (2.6)	0
**Benign**	4 (7)	1 (2.8)
Inflammatory with pathogen identified	1 (25)	0
Granulomatous	2 (50)	1 (100)
Organizing pneumonia	1 (25)	N/A

Data are presented as No. (%). RAB = robotic-assisted bronchoscopy; NSCLC = non-small cell lung cancer; NOS = not otherwise specified, N/A = not applicable.

**Table 4:** Factors associated with diagnostic yield between platforms.

		Univariable		Multivariable	
Variable	N	Odds Ratio (95% CI)	*p* Value	Adjusted Odds Ratio (95% CI)	*p* Value
Platforms	92				
Ion					
Monarch		0.87 (0.34–2.28)	0.77	0.89 (0.30–2.72)	0.83
Best radial ultrasound image acquisition	87				
Concentric					
Eccentric		0.47 (0.89–1.13)	0.13	0.46 (0.16–1.25)	0.13
Nodule size, mm	92	0.99 (0.89–1.13)	0.83	0.97 (0.85–1.11)	0.57
Location within lung thirds	92				
Central third					
Middle third		1.53 (0.38–5.96)	0.54	1.39 (0.31–5.94)	0.66
Peripheral third		1.87 (0.49–6.67)	0.34	1.73 (0.41–6.96)	0.44
Density of nodule	92				
Part solid					
Solid		0.33 (0.02–1.92)	0.30	0.30 (0.01–2.00)	0.29

CI = confidence interval.

## Data Availability

The data presented in this study are available on request from the corresponding author due to IRB requirements and pending IRB review.

## Abbreviations

CI	confidence interval
DT-ENB	digital tomosynthesis electromagnetic navigation bronchoscopy
ENB	electromagnetic navigation bronchoscopy
EMN-RAB	electromagnetic navigation robotic- assisted bronchoscopy
NSCLC	non-small cell lung carcinoma
PPL	peripheral pulmonary lesions
RAB	robotic-assisted bronchoscopy
REBUS	radial probe endobronchial ultrasound
TBNA	transbronchial needle aspiration
TTNA	transthoracic needle aspiration
